# Effect of high-fluoride toothpaste and mouth rinse on the prevention of demineralized lesions during orthodontic treatment: a randomized controlled trial

**DOI:** 10.1093/ejo/cjad044

**Published:** 2023-07-31

**Authors:** Hanna Enerbäck, Mai Lin Lövgren, Nicklas Strömberg, Anna Westerlund

**Affiliations:** Department of Orthodontics, Institute of Odontology, Sahlgrenska Academy, University of Gothenburg, Gothenburg, Sweden; Department of Orthodontics, Institute of Odontology, Sahlgrenska Academy, University of Gothenburg, Gothenburg, Sweden; Department of Cariology, Institute of Odontology, University of Umeå, Umeå, Sweden; Department of Orthodontics, Institute of Odontology, Sahlgrenska Academy, University of Gothenburg, Gothenburg, Sweden; Specialist Clinic for Orthodontics, Public Dental Service, Mölndal, Sweden

**Keywords:** fluoride, mouth rinse, high-fluoride toothpaste, white spot lesion, demineralization, fixed appliance, orthodontics, fixed brace treatment, multi bracket appliance treatment

## Abstract

**Objective:**

To evaluate the effect of high-fluoride mouth rinse and high-fluoride toothpaste on the development of demineralized lesions (DLs) during orthodontic treatment.

**Trial design:**

Three-armed parallel-group randomized controlled trial.

**Methods:**

The trial was performed with 270 adolescent orthodontic patients. Randomization was performed in blocks of 30, enrolling the patients into one of the following groups: the fluoride mouth rinse (FMR) group receiving 0.2% sodium fluoride (NaF) mouth rinse plus 1450 ppm fluoride (F) toothpaste; high-fluoride toothpaste (HFT) group receiving 5000 ppm F toothpaste; and the Control (CTR) group receiving 1450 ppm F toothpaste. Inclusion criteria were patients scheduled for treatment in both arches with fixed appliances and age between 12 and 20 years. The primary outcome variable was the proportion of participants with at least one new demineralized lesion as assessed on digital photos taken before and after treatment, analysed by a blinded clinician. The analysis included all teeth or teeth in the aesthetic zone, i.e. all central incisors, lateral incisors, and canines. A random sample of 30 participants was assessed to check intra- and inter-reliability. For pairwise comparison between groups, Fisher’s non-parametric permutation test was used for continuous variables. Blinding was employed during the caries registration and data analysis.

**Recruitment:**

October 2010 to December 2012

**Results:**

In total, 270 patients were randomized, of which 22 were excluded during treatment. Therefore, 248 participants were included in the study. The number of patients with an increase of ≥1 DL, including only central- and lateral incisors and canines, during orthodontic treatment, was significantly lower in the HFT group, 51/85 60%, compared to the CTR group, 64/82 78%, RR 0.77 (CI 0.62; 0.95), *P* = .01 and in the FMR group, 47/81 58%, compared to the CTR group, RR 0.74 (CI 0.60; 0.92), *P* < .01.

**Conclusions:**

To prevent demineralized lesions in the aesthetic zone, high-fluoride mouth rinse and high-fluoride toothpaste may be recommended.

**Limitations:**

The protocol was not registered, and the present study did not use a double-blinded design.

## Introduction

Early demineralized lesions (DLs), also called white spot lesions (WSLs), are a common and undesirable side effect of orthodontic treatment with fixed appliances [1–4]. Treatment with fixed appliances complicates oral hygiene measures. Levels of cariogenic bacteria increase [5–7], thereby promoting the accumulation of plaque [5, 8, 9] and an increased risk of early tooth decay. Three recently published RCTs involving 870 orthodontic patient participants, assessed from pre- and post-treatment photographs, found new demineralized lesions in around one-quarter of orthodontic patients at the end of treatment [10–12]. The lesions often appear on the front teeth and most frequently affect the maxillary lateral incisors [1, 10, 11, 13]. The DLs appear to be resistant to complete remineralization, which can cause aesthetic concern to the patient and jeopardize the final aesthetic outcome of the orthodontic treatment [3, 14]. Caries conservative and operative treatments may cause discomfort for the patient and are expensive and costly for the society.

There is good scientific support that fluoride complicates demineralization and facilitates remineralization [15], as well as affects the metabolism of the caries bacteria [16]. The preventive effects of fluoride toothpaste on caries are firmly established [17]. Various treatments of DL have been evaluated, including sealing with fluoride and casein solutions, resin infiltration, micro abrasion as well as bleaching, but there is a lack of reliable evidence-based research for these methods [18, 19]. To prevent DL during orthodontic treatment, the effectiveness of high-fluoride toothpaste on DL has been evaluated in a randomized clinical trial (RCT), demonstrating fewer and less severe DL [11]. A clinical study demonstrated a significant reduction in DLs in patients using mouth rinse (0.05% NaF) during orthodontic treatment [20] and an RCT demonstrated less demineralization in patients using fluoride rinse (150 ppm NaF and 100 ppm amine fluoride) compared to placebo rinse [21]. Systematic reviews indicate a positive effect of different topical fluoride products in addition to fluoride toothpaste [22, 23], but more high-quality trials are needed to increase the certainty of these findings and assess evidence-based guidelines [12].

High-fluoride mouth rinse (0.2% NaF) is commonly recommended to patients with an increased caries risk. To the author’s knowledge, no large RCTs with sufficient power have compared the effect of high-fluoride toothpaste (5000 ppm F) and high-fluoride mouth rinse (0.2% NaF) on the development of DL during orthodontics. The aim of the present study is to evaluate the effect of high-fluoride toothpaste and high-fluoride mouth rinse for preventing DL during orthodontic treatment. The null hypothesis was that there is no difference between the fluoride methods in preventing DL during orthodontic treatment.

## Subjects and methods

### Trial design

The single study employed a prospective randomized controlled clinical trial with three arms parallel group with 1:1:1 allocation ratio. The study design was approved by the Research Ethical Board in Gothenburg, Sweden (Reg. no. 321-09). The study protocol was not registered. No changes were made to the methods after trial commencement.

### Participants, eligibility criteria, and settings

Recruitment for the study started in October 2010 and continued to December 2012. In total, 300 participants who were referred to the Specialist Clinic for Orthodontic Dentistry, Public Dental Service (Mölndal, Sweden) were invited to participate in the study. The study population for the study was patients’ residents to one of nine different Public Dental Service Clinics. Five experienced orthodontists were involved in the orthodontic treatment. Inclusion criteria of the study were age between 12 and 20 years and scheduled for orthodontic treatment of both the upper and lower arches with fixed appliances (MBTTM (McLaughlin, Bennett, Trevisi), pre-adjusted with 0.022-inch slots; 3M Unitek Orthodontic Products, Monrovia, CA, USA) for an expected treatment duration of at least 1 year. The exclusion criteria were treatment with a removable appliance or a lingual fixed appliance and patients with severe disease. Severe disease was defined as patients who, due to their health, were not suitable for general dental care. The patients received information both verbally and in writing regarding the study. After agreeing to participate, each patient signed an informed consent form (or the caregiver signed if the patient was <18 years of age). All the participants lived in communities with a natural fluoride level of < 0.1 mg/L in tap water.

### Interventions

Patients were randomly allocated to one of the following groups with associated fluoride protocols:

#### Fluoride mouth rinse (FMR) group.

Rinsing with a fluoride mouth rinse (0.2% NaF, corresponding to 900 ppm F, Flux; Actavis, Stockholm, Sweden) twice daily and tooth brushing twice a day; using standard toothpaste (1450 ppm F, Colgate Caries Control; Colgate-Palmolive, Lyngby, Denmark). The patients were instructed to use the provided fluoride mouth rinse after brushing their teeth.

#### High-concentration fluoride toothpaste (HFT) group.

Tooth brushing twice daily using a high-concentration fluoride toothpaste (5000 ppm F, Duraphat; Colgate-Palmolive).

#### Fluoride toothpaste (FT) control group.

Tooth brushing twice daily using standard toothpaste (1450 ppm F, Colgate Caries Control; Colgate-Palmolive).

The participants were given verbal and written information regarding product use. The patients were instructed to apply 2 cm (approximately 1 g) of toothpaste to the brush, in accordance with the manufacturer’s instructions. The recommended tooth brushing time was 2 min, performed after breakfast and before going to bed. The FMR group was instructed to rinse for 2 min with 10 ml of the fluoride mouth rinse. Patients were instructed not to use water during or after brushing and rinsing and to avoid intake of food and drink for at least 1 h after tooth brushing and mouth rinsing. The patients were also supplied with a toothbrush (Lactona, Bergen op Zoom, Netherlands) every third month, to be used throughout the treatment period. The fluoride products were provided free of charge to the patients, and the patient’s compliance to the products was evaluated in a questionnaire.

### Outcomes

The predictor variables in this study were the use of different fluoride products. The primary outcome variable was the DL incidence; the proportion of participants with at least one new demineralized lesion, as assessed on digital photos taken before and after orthodontic treatment on the buccal surfaces of permanent teeth. Aesthetic impact (degree of injury from 1 to 4) was assessed as secondary outcomes. No changes were made to the trial outcomes after trial commencement. Before and after treatment, standard intraoral close-up photographs (one frontal photograph and two side photographs) were taken with a digital camera (Canon Powershot G7X; Canon, Inc., Tokyo, Japan) and stored in the patient’s digital journal (Edward). The photographs were taken before bonding, at the same visit as the bonding took place. The buccal surfaces of both of the maxillary and mandibular incisors, canines, premolars, and first molar were included in the DL registration. The photographs were taken ‘edge-to-edge’. The DL was assessed according to a Gorelick *et al*. score [1], 1 = no white spot formation, 2 = slight white spot formation (thin rim), 3 = excessive white spot formation, and 4 = white spot formation with cavitation. Teeth that were extracted during orthodontic treatment were excluded from the pre- and post-values. Teeth presented with environmental and developmental alterations, such as enamel hypoplasia, fluorosis, and stains, were excluded.

At baseline, the teeth were polished with a rubber cup and pumice paste and gently dried before photographs were taken. At the time of debonding, the composite material on the teeth surfaces was carefully removed with a slowly rotating carbide bur, followed by polishing with a rubber cup. After gently air drying, a new series of frontal and lateral digital photos were taken. The photographs were projected on a screen (Elite Display E222; Hewlett Packard, Palo Alto, CA, USA) in a dark room while one of the authors (HE) assessed DL. If in doubt when scoring, the lower score was chosen. Missing or poor photos were excluded from the analysis.

In order to establish compliance regarding fluoride intake during treatment (at 1 year after installation of the fixed appliance), all the participants were asked to respond in writing to a questionnaire. The following questions were included in the questionnaire: *How often do you brush your teeth? How often do you use toothpaste? Do you use any additional fluoride product?* High-fluoride toothpaste and mouth rinse were counted as additional fluoride products. The patients were also asked about their general disease status, if they had any disease(s), and if they used any medications.

### Sample size calculation

The sample size estimation was determined with a power calculation assessing superiority, with the significance level set at 0.05 and 80% power. With the α and β values set at 0.05 and 0.2, respectively, 66 patients per group were needed to disclose a difference of 25% between the groups in the proportion of patients with an increase of ≥1 DL during orthodontic treatment. With an expected attrition rate of 15%, a total number of 76 in each group was considered to be sufficient.

### Interim analyses and stopping guidelines

Not applicable.

### Randomization and blinding

In this single-blind trial, a randomization sequence was generated in blocks of 30 to ensure that equal numbers of patients were allocated to each group. Thirty paper sheets (10 FMR, 10 HFT, and 10 CTR) were folded and placed in a basket (AW). Before treatment commenced, each patient selected a paper sheet from the basket for randomization. Until the moment of assignment, the allocation sequence was concealed from those assigning participants to the intervention groups. The orthodontists and the orthodontic assistants enrolled participants to their intervention group. The author assessing DL was not involved in the clinical data collection or in the treatment of the patients and was blinded to the patient’s group allocation. The patient’s group affiliation was revealed after the statistical analysis.

### Statistical analysis

Statistical analysis was performed using the Statistical Package for Social Sciences (SPSS® version 20.0 for Windows, SPSS, Inc., Chicago, IL, USA) and SAS 9.4 (SAS 9.4 by SAS Institute, Inc., Cary, NC, USA). For continuous variables, mean (SD)/median (min; max)/number are presented. For categorical variables, *n* (%) is presented. For comparison between groups, Fisher’s Exact test was used for dichotomous variables, and the Mantel-Haenszel chi-square test was used for ordered categorical variables. Adjustment for variable baseline values was made as well as treatment time (months) using logistic regression. For comparison between groups, the Kruskal–Wallis test was used for continuous variables. For pairwise comparison between groups, the Mann–Whitney *U*-test was used for continuous variables. To assess reliability, all photographs were reassessed 1 month later by the author (HE), and a random collection of photographs for 30 patients was assessed by another experienced dentist (ML). Cohen’s κ and weighted κ (on a scale from 0 to 2 on each tooth surface) were calculated to assess intra and inter-examiner agreement.

## Results

### Participants and baseline data

**Figure 1.**
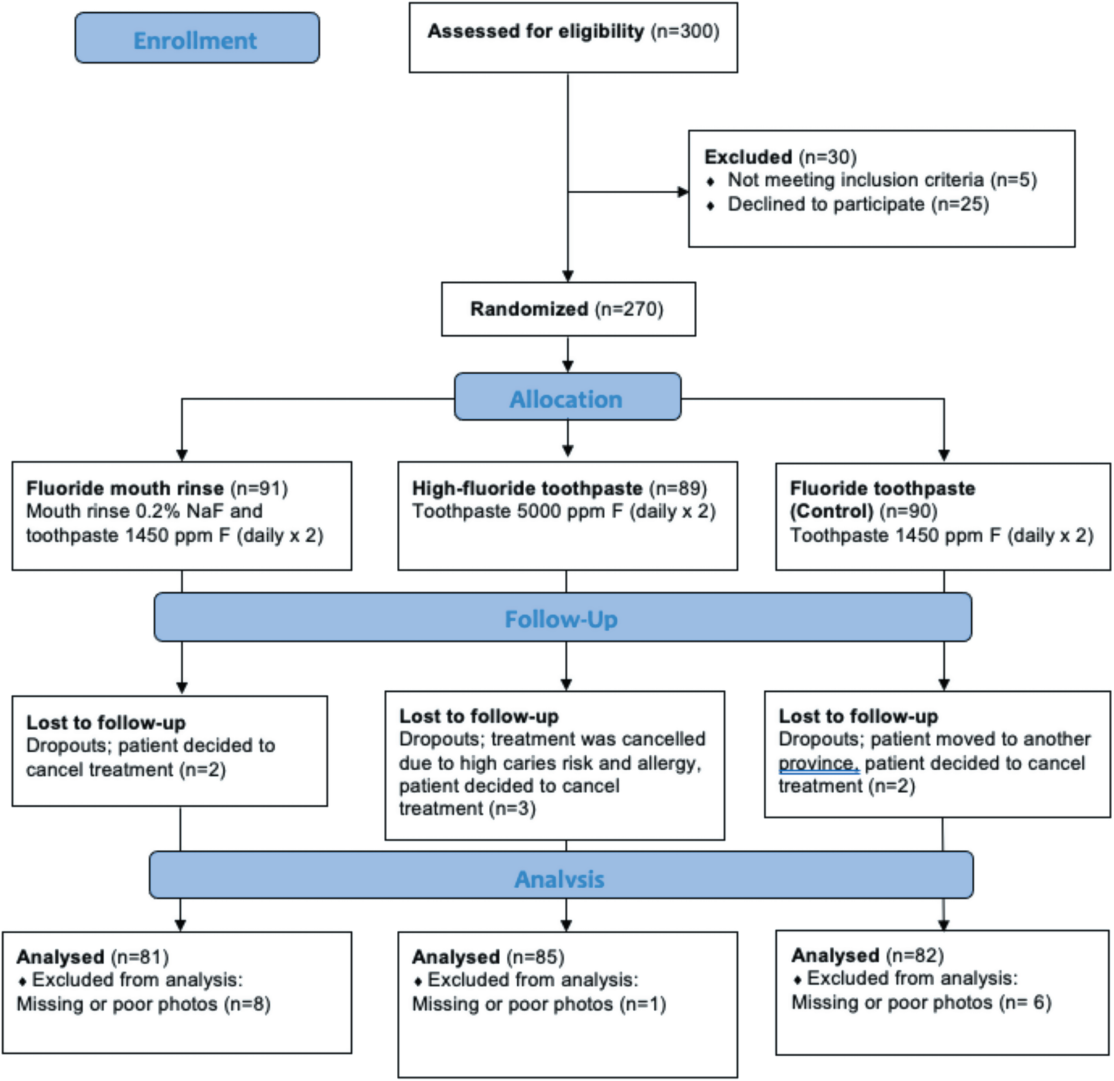
Flowchart for participants and dropouts in the trial. Adapted from CONSORT.

In total, 270 participants were included in the study, of which 95 were males and 175 were females. The dropout rate was 8.1% (22 patients), including seven patients who were lost to follow-up due to several reasons (Fig. 1). Another 15 patients were excluded from the analysis due to incomplete or poor photos. Therefore, 248 patients with complete photos (before and after treatment) were included in the study.

At baseline, the gender distribution for the 248 patients was 84 (33.9%) males and 164 (66.1%) females. A similar distribution was also seen between the three intervention groups (Table 1). The mean patient age at baseline was 15.4 (SD 1.6), and 30 participants stated a disease, with asthma and/or allergy being the most prevalent (Table 1). The mean orthodontic treatment duration was 25.9 (± 9.1) months, i.e. 25.0 (±9.3) months for the FMR group, 27.8 (± 10.3) months for the HFT group, and 24.7 (±7.3) months for the CTR group.

**Table 1.** Distribution of sex, age, and health conditions between the groups at baseline.

	Fluoride mouthrinse (*n* = 81)	High-fluoride toothpaste(*n* = 85)	Fluoride toothpaste(control) (*n* = 82)
Sex			
* Male (*n*, %)*	27 (33%)	30 (35%)	27 (33%)
* Female (*n*, %)*	54 (67%)	55 (65%)	55 (67%)
Age			
*Mean (SD)*	15.1 (1.6)	15.6 (1.6)	15.6 (1.7)
Health			
* No disease (*n*, %)*	68 (84.0)	74 (87.1)	76 (92.7)
* Asthma and/or allergy*	7 (8.6)	10 (11.8)	5 (6.1)
* Epilepsy*	1 (1.2)		1 (1.2)
* Muscle disorder*		1 (1.2)	
* Migraine*	1 (1.2)		
* Depression, anxiety*	1 (1.2)		
* Autism*	1 (1.2)		
* ADHD*	2 (2.5)		

### DL on the patient level

At baseline, 17 patients (21.0%) in the FMR group showed 0 DL, while 22 patients (25.9%) in the HFT group and 15 (18.3%) patients in the control group showed 0 DL. There was no statistically significant difference between the groups regarding baseline DL status on patient level (Table 3). Patients with an increase of ≥1 DL did not differ significantly between the groups during orthodontic treatment, when all teeth were included in the analysis. Furthermore, no differences were seen between the groups regarding the number of baseline DL when only taking into account the teeth in the ‘aesthetic zone’, including all central incisors, lateral incisors, and canines in the upper and lower arches. However, the number of patients with an increase of ≥1 DL in the ‘aesthetic zone’, during orthodontic treatment, was significantly lower in the HFT group, 51/85 60%, compared to the CTR group, 64/82 78%, RR 0.77 (CI 0.62; 0.95), *P* = .01 and in the FMR group, 47/81 58%, compared to the CTR group, RR 0.74 (CI 0.60; 0.92), *P* < .01; Tables 2 and 3, Supplementary Fig. 1). The differences between the two intervention groups were not statistically significant (*P* = .67).

**Table 2.** Relative risks (risk ratios) for increase in ≥1, ≥2, and ≥3 demineralized lesion (DL) in all teeth and in the aesthetic zone, pairwise comparisons with 95% CI. Unadjusted values.

		RR (95% CI)		
		HFT vs CTR	FMR vs CTR	FMR vs HFT
Teeth	Increase in WSL			
All teeth	≥1 WSL	0.93 (0.77; 1.13)	0.89 (0.73; 1.10)	0.96 (0.77; 1.19)
	≥2 WSL	0.93 (0.72; 1.19)	0.85 (0.65; 1.12)	0.92 (0.69; 1.22)
	≥3 WSL	0.85 (0.61; 1.18)	0.72 (0.50; 1.03)	0.85 (0.58; 1.24)
Aesthetic zone	≥1 WSL	0.77 (0.62; 0.95)	0.74 (0.60; 0.92)	0.97 (0.75; 1.25)
	≥2 WSL	0.88 (0.67; 1.16)	0.82 (0.62; 1.10)	0.93 (0.69; 1.26)
	≥3 WSL	0.89 (0.62; 1.26)	0.79 (0.54; 1.16)	0.90 (0.61; 1.32)

**Table 3.** The table presents demineralized lesions (DLs) on patient level, *n* (%) and between-group comparisons.

	Fluoride mouth rinse (*n* = 81)	High-fluoride toothpaste (*n* = 85)	Control (*n* = 82)	*P*-value*		
				MR vs HFT	CTR vs HFT	CTR vs FMR
All teeth included						
WSL pre-treatment				.76	.41	.42
0	17 (21.0%)	22 (25.9%)	15 (18.3%)			
1–3	33 (40.7%)	35 (41.2%)	37 (45.1%)			
4–8	28 (34.6%)	24 (28.2%)	21 (25.6%)			
≥9	3 (3.7%)	4 (4.7%)	9 (11.0%)			
WSL post-treatment						
0	12 (14.8%)	7 (8.2%)	6 (7.3%)	.90	.21	.21
1–3	19 (23.5%)	25 (29.4%)	22 (26.8%)			
4–8	31 (38.3%)	34 (40.0%)	28 (34.1%)			
≥9	19 (23.5%)	19 (22.4%)	26 (31.7%)			
Patients with an increase of						
≥1 WSL	53 (65.4%)	58 (68.2%)	60 (73.2%)	.71	.37	.28
≥2 WSL	42 (51.9%)	48 (56.5%)	50 (61.0%)	.60	.42	.25
≥3 WSL	29 (35.8%)	36 (42.4%)	41 (50.0%)	.41	.23	.076
Teeth in the esthetic zone included						
WSL pre-treatment						
0	59 (72.8%)	59 (69.4%)	58 (70.7%)	.23	.48	.42
1–3	21 (25.9%)	22 (25.9%)	22 (26.8%)			
≥4	1 (1.2%)	4 (4.7%)	2 (2.4%)			
WSL post-treatment						
0	23 (28.4%)	26 (30.6%)	15 (18.3%)	.71	.12	.054
1–3	33 (40.7%)	28 (32.9%)	31 (37.8%)			
4–8	25 (30.9%)	31 (36.5%)	36 (43.9%)			
Patients with an increase of						
≥1 WSL	47 (58.0%)	51 (60.0%)	64 (78.0%)	.67	.011	.0059
≥2 WSL	39 (48.1%)	44 (51.8%)	48 (58.5%)	.57	.31	.19
≥3 WSL	29 (35.8%)	34 (40.0%)	37 (45.1%)	.66	.39	.25

Aesthetic zone includes teeth 3-3 in the maxillary and mandibular arches. *P*-value* is adjusted for baseline and treatment time.

### DL based on surface level and lesion severity

In total, 5465 buccal tooth surfaces were scored before and after baseline. At baseline, the first molar was most frequently affected by DL, 163 (65.7%) patients showed DL on one, two, three or all first molars. During treatment, the central incisor, lateral incisors, and canins in the upper arch demonstrated the highest frequency of DL. During treatment, 94 patients (37.9%) showed DL on the right central incisor and 107 (43.1%) on the left central incisor, the corresponding figures for the lateral incisor and canines were 99 (39.9%), 94 (37.9%), and 38 (15.3%) and 35 (14.1%) (Supplementary Table 1). Regarding the degree of injury, the distribution did not differ statistically significant between the groups (Table 4).

**Table 4.** The distribution (*n* %) of aesthetic impact [1–4], on surface level after orthodontic treatment.

Group score	FMR (*n* = 1944)	HFT (*n* = 2040)	CTR (*n* = 1968)
1	1374 (76.0%)	1408 (75.6%)	1273 (70.8%)
2	391 (21.6%)	404 (21.7%)	482 (26.8%)
3	42 (2.3%)	51 (2.7%)	41(2.3%)
4	0 (0%)	0 (0%)	1 (0.1%)

### Intra- and inter-examiner reliability levels

Cohen’s κ and weighted κ values were calculated to determine intra- (reassessment of the collection after 1 month) and inter-examiner reliability (two examiners assessed photographs from 30 patients) for DL measurements. The intra-examiner κ value was 0.81 (very good), and the inter-examiner κ value was 0.78 (good). Intra-rater weighted κ showed 0.81 (95% CI 0.78–0.84), and inter-rater weighted κ showed 0.78 (95% CI 0.75–0.81).

### Compliance

Compliance was evaluated 1 year after the initiation of treatment. Of the participants, 236 (95.2%) of the 248 participants stated that they brushed their teeth at least twice a day during the treatment. All patients stated that they used a fluoride toothpaste for brushing. In addition, 76 patients (93.8%) in the FMR group and 79 patients (92.4%) in the HFT group answered that they used additional high-fluoride products.

### Harms

No patient reported any adverse events, such as allergy or other harms, in relation to using the fluoride products.

## Discussion

The number of patients with an increase of ≥1 DL during orthodontic treatment was significantly higher in the CTR group compared to the HFT group and the FMR group when only central incisors, lateral incisors, and canines were included in the analysis. Therefore, in the aesthetic zone, patients benefitted from using fluoride mouth rinse and high-fluoride toothpaste compared to ordinary fluoride toothpaste. The positive effect of high-fluoride toothpaste and fluoride rinse on the development of DL are in line with the findings of previous clinical studies [11, 20, 21]. However, no effect was seen on the patient level when all the teeth were included. Several clinical studies with similar study designs do not take into account the first molar in their analyses [10, 11, 13]. The first molars were banded during treatment, a factor that may influence the development of DLs. Moreover, from an aesthetic point of view, the appearance of the front teeth is probably most interesting for the patient. There are several different ways to administrate fluoride. Recently published RCTs have been showing promising effects on preventing demineralization during orthodontic treatment for HFT [11], FMR [21], and fluoride varnish [10, 24]. However, the number of teeth evaluated in these studies vary, and the preventive effect on the central incisors, lateral incisors, and canines during orthodontic treatment has not before been thoroughly evaluated. Another factor to consider is the economics of the products used; high-fluoride toothpaste is significantly more costly than fluoride mouth rinse. However, in some countries, as in Sweden, the high-fluoride toothpaste is included in the high-cost cover, which makes the price difference between mouth rinse and high-fluoride tooth paste comparable. In this present study, the patients were given the products free of charge. However, when patients are expected to pay, the compliance may be affected. The prevalence of demineralized lesions is high in this study compared to other RCTs in this field [10–12]. Potential explanations may be the number of teeth analysed, diagnosis of the lesion (dochotomous value or degree of injury), the number of assessors, and the equipment used for determining demineralization (such as camera equipment and computer screen).

DLs can persist and create aesthetic concerns for the patient even 12 years after treatment [3]. In line with the present study, other clinical studies present that the lateral incisor in the upper arch is frequently affected by DL [1, 13, 14, 20, 25]. Explanatory factors may be attributed to higher plaque accumulation due to palatinal positioned lateral incisors that complicate oral hygiene measures. Another more hypothetical theory is the lack of saliva access. In the present study, the teeth in the aesthetic zone showed the highest incidence of DLs during treatment, indicating a better effect on high-fluoride products on caries-prone teeth. The DLs were in favour of the high-fluoride interventions, and the null hypothesis could partly be rejected. In line with the findings, it would be interesting, in future studies, to develop a new tooth index, taking into account teeth in the aesthetic zone, including particularly caries-prone surfaces in order to more effectively target and evaluate the effect of different caries prophylaxis.

This study showed a higher baseline prevalence of DL (proportion of patients with at least one white spot lesion) compared to studies with a similar study design [10, 11]. However, the prevalence of DL varies greatly between studies, 2–96% [1–4, 26], which can be explained by the variation in the number of teeth included in the analysis, the patient’s caries risk, the patient’s compliance to the fluoride product used and differences in diagnostic methods. However, scoring DL on digital photos has been shown to be a method comparable to clinical examination [13, 27]. Moreover, we demonstrate high intra-reliability (κ values 0.81) when a sample of 30 photos was reassessed a month later by the same dentist, as well as high inter-reliability (κ values 0.78) when two dentists assessed the same collection of 30 photos. It can be discussed if several independent observers would have further improved the assessment of demineralization. A recent RCT found that five assessors were required to achieve a majority consensus on the presence or absence of demineralized lesions from photographs of all the participants in the trial [12].

A weakness of the present study is that the randomization was performed manually with sealed envelopes. It would have been preferable to randomize the patients using computer-generated sequence numbers. Another study limitation is that the present study did not use a double-blinded design. The protocol was not published before trial commencement, which must be seen as a weakness. The study was planned 15 years ago, and at that time, trial registration was not a requirement as it is today. Furthermore, patients could have been prescribed additional fluoride products from their general dentist at their home clinics, which could have affected the patient’s DL status during treatment. However, there are several strengths of the study. The study had a randomized controlled trial design ensuring high scientific value. Furthermore, the assessor of the DL score was blinded to the patient’s group affiliation and not involved in the clinical procedure, which minimized the risk for bias. The large material size, sufficient power, and the fact that all the patients were provided with the same toothbrush and fluoride products are strengths of the present study. Furthermore, the good intra- and inter-rater agreement also strengthened the findings of this study.

### Generalizability

The study findings can be generalized for patient groups with similar baseline DL status, mean age, inclusion/exclusion criteria, and treatment protocols. However, single centre reduces generalizability, albeit with multiple operators.

## Conclusions

A positive effect of high fluoride toothpaste and fluoride mouth rinse in preventing DL on central incisors, lateral incisors, and canines during orthodontic treatment. DLs are still a problem during orthodontic treatment. Therefore, future randomized controlled clinical trials are needed to assess evidence-based guidelines for preventing DL during orthodontic treatment.

## Supplementary Material

cjad044_suppl_Supplementary_Figure_S1

cjad044_suppl_Supplementary_Table_S1

## Data Availability

Data are available on request.
