# Supplementary figures and images for: Effect of high-fluoride toothpaste and mouth rinse on the prevention of demineralized lesions during orthodontic treatment: a randomized controlled trial
Source: Eur J Orthod. 2023 Jul 31;45(5):477–84. doi: 10.1093/ejo/cjad044 (PMC10566543; doi:10.1093/ejo/cjad044)

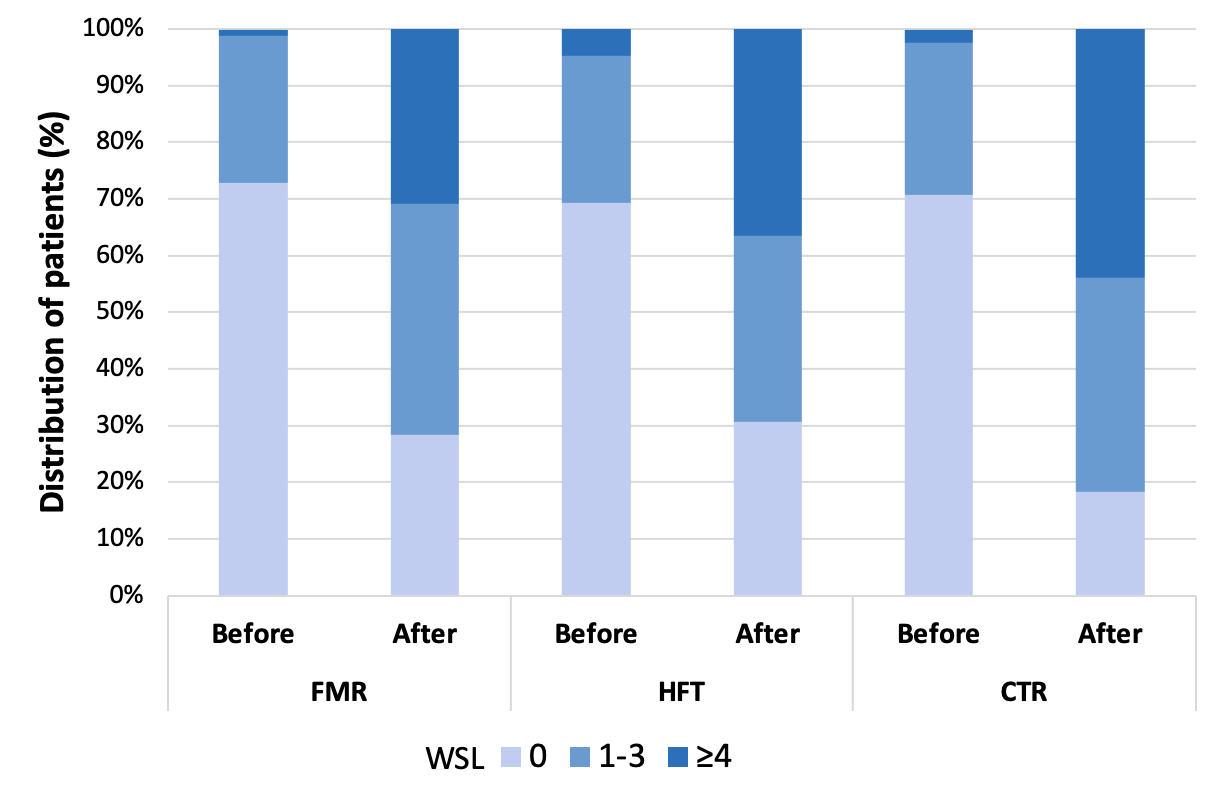

Supplement: cjad044_suppl_Supplementary_Figure_S1 [file cjad044_suppl_supplementary_figure_s1.docx]
