# Supplementary material for: Effect of high-fluoride toothpaste and mouth rinse on the prevention of demineralized lesions during orthodontic treatment: a randomized controlled trial
Source: Eur J Orthod. 2023 Jul 31;45(5):477–84. doi: 10.1093/ejo/cjad044 (PMC10566543; doi:10.1093/ejo/cjad044)
Supplement: cjad044_suppl_Supplementary_Table_S1 [file cjad044_suppl_supplementary_table_s1.docx]

| **Tooth**  **WSL** | **16** | **15** | **14** | **13** | **12** | **11** | **21** | **22** | **23** | **24** | **25** | **26** |
| --- | --- | --- | --- | --- | --- | --- | --- | --- | --- | --- | --- | --- |
| Before | 95 (38.6%) | 45 (20.4%) | 18 (12.0%) | 18 (7.3%) | 8 (3.2%) | 15 (6.0%) | 16 (6.5%) | 13 (5.2%) | 23 (9.4%) | 18 (11.9%) | 43 (19.8%) | 118 (48.2%) |
| After | 96 (39.0%) | 57 (25.8%) | 26 (17.3%) | 56 (22.8%) | 107 (43.1%) | 109 (44.0%) | 123 (49.6%) | 107 (43.1%) | 58 (23.7%) | 32 (21.2%) | 62 (28.6%) | 116 (47.3%) |
| ΔChange: |  | | | | | | | | | | | |
| Decrease | 24 (9.8%) | 9 (4.1%) | 5 (3.3%) | 9 (3.7%) | 2 (0.8%) | 1 (0.4%) | 1 (0.4%) | 1 (0.4%) | 4 (1.6%) | 4 (2.6%) | 5 (2.3%) | 27 (11.0%) |
| Same | 197 (80.1%) | 191 (86.4%) | 132 (88.0%) | 190 (77.2%) | 145 (58.5%) | 152 (61.3%) | 139 (56.0%) | 152 (61.3%) | 202 (82.4%) | 129 (85.4%) | 188 (86.6%) | 193 (78.8%) |
| Increase | 25 (10.2%) | 21 (9.5%) | 13 (8.7%) | 47 (19.1%) | 101 (40.7%) | 95 (38.3%) | 108 (43.5%) | 95 (38.3%) | 39 (15.9%) | 18 (11.9%) | 24 (11.1%) | 25 (10.2%) |
| **Tooth**  **WSL** | **46** | **45** | **44** | **43** | **42** | **41** | **31** | **32** | **33** | **34** | **35** | **36** |
| Before | 81 (32.9%) | 25 (13.2%) | 26 (13.3%) | 12 (4.8%) | 9 (3.6%) | 4 (1.6%) | 4 (1.6%) | 5 (2.0%) | 11 (4.5%) | 21 (10.9%) | 28 (14.2%) | 85 (34.7%) |
| After | 89 (36.2%) | 32 (16.9%) | 39 (20.0%) | 39 (15.7%) | 25 (10.1%) | 19 (7.7%) | 18 (7.3%) | 15 (6.0%) | 30 (12.1%) | 36 (18.8%) | 31 (15.8%) | 90 (36.7%) |
| ΔChange: |  | | | | | | | | | | | |
| Decrease | 19 (7.7%) | 7 (3.7%) | 5 (2.6%) | 2 (0.8%) | 4 (1.6%) | 2 (0.8%) | 2 (0.8%) | 4 (1.6%) | 4 (1.6%) | 8 (4.2%) | 10 (5.1%) | 18 (7.3%) |
| Same | 200 (81.3%) | 168 (88.9%) | 172 (88.2%) | 217 (87.5%) | 224 (90.3%) | 229 (92.3%) | 230 (92.7%) | 230 (92.7%) | 220 (89.1%) | 161 (83.9%) | 173 (88.3%) | 204 (83.3%) |
| Increase | 27 (11.0%) | 14 (7.4%) | 18 (9.2%) | 29 (11.7%) | 20 (8.1%) | 17 (6.9%) | 16 (6.5%) | 14 (5.6%) | 23 (9.3%) | 23 (12.0%) | 13 (6.6%) | 23 (9.4%) |
